# Cultured corneas show dendritic spread and restrict herpes simplex virus infection that is not observed with cultured corneal cells

**DOI:** 10.1038/srep42559

**Published:** 2017-02-15

**Authors:** Neel Thakkar, Dinesh Jaishankar, Alex Agelidis, Tejabhiram Yadavalli, Kyle Mangano, Shrey Patel, Sati Zeynep Tekin, Deepak Shukla

**Affiliations:** 1 Department of Ophthalmology and Visual Sciences, University of Illinois at Chicago, IL 60612, USA; 2 Department of Bioengineering, University of Illinois at Chicago, IL 60612, USA; 3 Department of Microbiology and Immunology, University of Illinois at Chicago, IL 60612, USA; 4 Department of Medicinal Chemistry and Pharmacognosy, University of Illinois at Chicago, IL 60612, USA; 5 Department of Microbiology, Canakkale Onsekiz Mart University, Canakkale 17020, Turkey

## Abstract

Herpes simplex virus-1 (HSV-1) causes life-long morbidities in humans. While fever blisters are more common, occasionally the cornea is infected resulting in vision loss. A very intriguing aspect of HSV-1 corneal infection is that the virus spread is normally restricted to only a small fraction of cells on the corneal surface that connect with each other in a dendritic fashion. Here, to develop a comprehensive understanding of the susceptibility of human corneal epithelial (HCE) cells to HSV-1 infection, we infected HCE cells at three different dosages of HSV-1 and measured the outcomes in terms of viral entry, gene and protein expression, viral replication and cytokine induction. In cultured cells, infectivity and cytokine induction were observed even at the minimum viral dosage tested, while a more pronounced dose-restricted infectivity was seen in *ex vivo* cultures of porcine corneas. Use of fluorescent HSV-1 virions demonstrated a pattern of viral spread *ex vivo* that mimics clinical findings. We conclude that HCE cell cultures are highly susceptible to infection whereas the cultured corneas demonstrate a higher ability to restrict the infection even in the absence of systemic immune system. The restriction is helped in part by local interferon response and the unique cellular architecture of the cornea.

Herpes simplex virus (HSV) belongs to the *Herpesviridae* family, which is subdivided into alpha, beta and gamma subfamilies^1^. All *Herpesviridae* contain a double-stranded DNA genome, a proteinaceous capsid, which protects the viral genome, and a lipid bilayer containing glycoproteins that encapsulate the capsid^2^. HSV-1 and -2 are members of the alpha subfamily of herpesviruses and are highly ubiquitous human pathogens that establish life-long latent infections in their hosts^3^. HSV-1 is known to cause vesicular lesions of the oral region and keratitis of the eye and is one of the leading causes of corneal blindness^4^,^5^,^6^.

Herpetic infection of the eye and more specifically, the cornea, an immune privileged tissue, poses a unique challenge in our quest to study corneal infection by HSV-1 and develop new therapeutic tools to combat it. The corneal surface is constantly exposed to infectious pathogens and to maintain its avascularity and immune privileged status it must try to thwart infections with or without the involvement of systemic immune mechanisms^7^,^8^. In this regard, the corneal epithelial cells constitute the first line of defense against HSV-1, and these cells respond to infection by signaling the release of proinflammatory cytokines to recruit neutrophils and mononuclear lymphocytes into the cornea^9^. In addition, production of factors such as type 1 interferons (IFNs) can enhance the antiviral activity of the corneal epithelial cells. A unique combination of both mechanisms is thought to restrict the virus spread and its cytolytic effects to only certain cells of the corneal surface that connect with each other in a dendritic fashion^10^.

While it is clear that cultured human corneal epithelial (HCE) cells can be infected with HSV-1^2^,^11^,^12^,^13^,^14^,^15^, no comprehensive knowledge exists on the use of these cells for the study of a productive infection by HSV-1 and likewise, it remains unclear whether infection of HCE cells by HSV-1 reflects the uniqueness seen with the corneal infection. It is also unclear whether the cytokine response has any connection with the MOI of viral intruders. The latter could be crucial for controlling the disease and also for signaling the influx of immune cells^16^. Finally, it is unclear whether the highly restricted spread of the infection in the cornea is due to the involvement of the immune system or the cornea itself having an inherent ability to restrict the infection. To address the above-mentioned issues, this study generates a comprehensive analysis of the *in vitro* susceptibility of HCE cells to a range of HSV-1 virions and also confirms the findings using an *ex vivo* model of the cultured porcine cornea. Our results show that *in vitro* cultures of HCE cells can be infected with a range of viral dosages, all resulting in new virion production. The extent of cytokine response also shows a very interesting dependence on the initial virus titers used for infection. We demonstrate that cultured corneas are capable of restricting the infection and can provide an excellent model for studying the uniqueness of virus spread in the eye and testing the effects of antiviral drugs.

## Results

### HSV-1 enters into HCE cells in a dose-dependent manner

**Figure 1:**
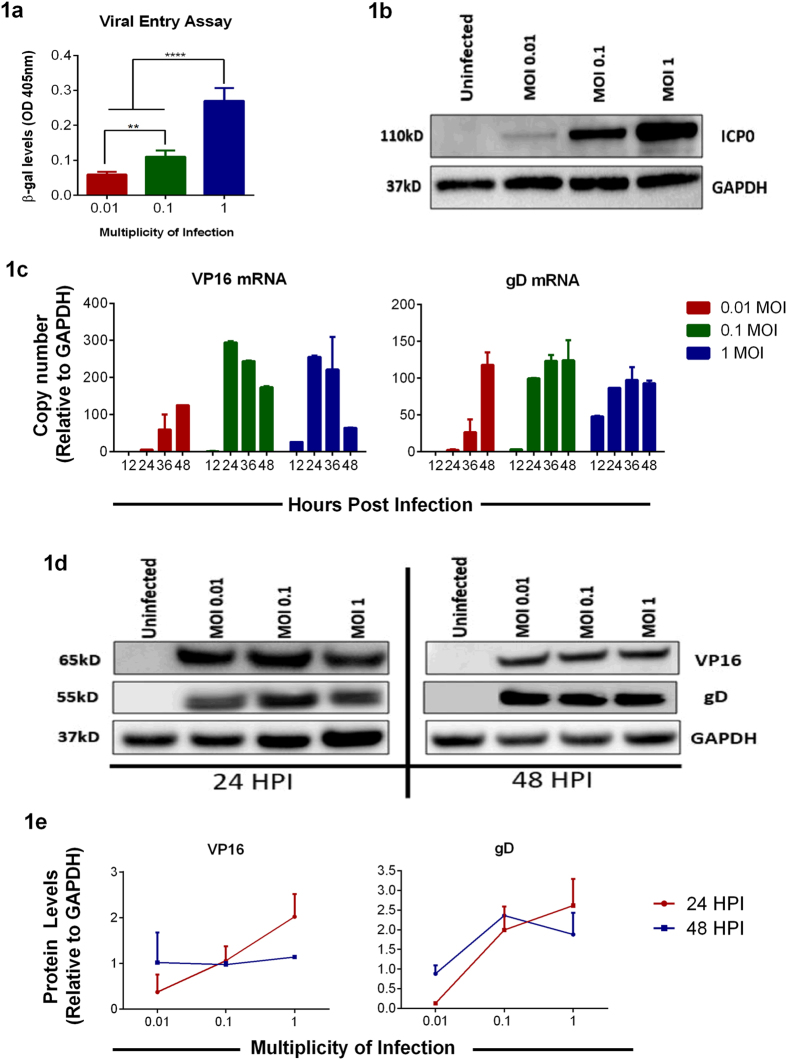
Viral entry and gene expression in HCE cells. **(a)** Entry assay using a recombinant virus was performed on HCE cells as mentioned in Materials and Methods. The color was read at 410 nm. *Asterisks* denote a significant difference determined by one-way ANOVA and multiple comparisons with Tukey’s correction: ****P < 0.0001, ***P < 0.001, **P < 0.01, *P < 0.05. **(b)** HCE cells in 6 well plates were infected with HSV-1 KOS at indicated MOIs. 6 hpi, samples were harvested, lysed and immunoblotted to assess the ICP0 levels. Representative blot is shown. **(c)** HCE cells were infected with HSV-1 KOS at indicated MOIs. At respective time points, RNA was extracted and cDNA was prepared as mentioned under Materials and Methods. qRT-PCR with primers specific to reported viral transcript was performed. Relative levels to GAPDH are shown. Two-way ANOVA statistical analysis with Tukey correction was performed to determine the significance. 12 hpi: 1.0 vs 0.1, ns; 1.0 vs 0.01, ns; 0.1 vs 0.01, ns. 24 hpi: 1.0 vs 0.1, ns; 1.0 vs 0.01, ****P < 0.0001; 0.1 vs 0.01, ****P < 0.0001. 36 hpi: 1.0 vs 0.1, ns; 1.0 vs 0.01, ***P < 0.001; 0.1 vs 0.01, ****P < 0.0001. 48 hpi: 1.0 vs 0.1, **P < 0.01; 1.0 vs 0.01, ns; 0.1 vs 0.01, ns. **(d)** A representative immunoblot showing viral protein levels for VP16 and gD from HCE cells at indicated times is shown. **(e)** Protein expression levels were quantified from three independent experiments using ImageJ software. Two-way ANOVA was performed using Tukey correction. **VP16**: 24 hpi: 0.01 vs 0.1, ns; 0.01 vs 1.0, *P < 0.05; 0.1 vs 1.0, ns; 48 hpi: 0.01 vs 0.1, ns; 0.01 vs 1.0, ns; 0.1 vs 1.0, ns. **gD**: 24 hpi: 0.01 vs 0.1, *P < 0.05; 0.01 vs 1.0, **P < 0.01; 0.1 vs 1.0, ns; 48 hpi: 0.01 vs 0.1, *P < 0.05; 0.01 vs 1.0, ns; 0.1 vs 1.0, ns.

Entry into cells is the first step of HSV-1 lifecycle^14^,^17^. To determine the amount of virion particles entering cells, a reporter-based entry assay was performed as described previously^18^. Using a recombinant HSV-1 (KOS) gL86 virus that encodes a β-galactosidase gene^19^ at MOIs 1, 0.1 and 0.01, we observed that viral entry at MOI 1 was the highest and the least amount of viral entry was observed with MOI 0.01 in HCE cells (Fig. 1a). To further confirm that viral entry is MOI-dependent, HCE cells were infected with HSV-1 (KOS) at MOIs 1, 0.1 and 0.01 for 6 hrs and immunoblotted for infected cell protein (ICP0), an early viral gene product that is transcribed upon entry. In agreement with the reporter-based entry assay, we observed that ICP0 protein levels were the highest at MOI 1 and lowest at MOI 0.01 (Fig. 1b). Taken together, these results indicate that even the lowest titers of HSV-1 virions can enter HCE cells.

### HSV-1 transcript and protein levels vary with MOI and time

We then proceeded to determine HSV-1 transcription and protein levels in HCE cells^20^. HCE cells were infected at three different MOIs and at indicated times cells were collected and levels of viral transcripts were quantified via qRT-PCR^21^. In general, infection at MOI 1 showed an initial increase in viral transcripts compared to MOIs 0.01 and 0.1. However, as infection progressed, viral transcripts at this MOI seemed to increase slightly and diminished at 48 hpi. On the contrary, a robust production of viral transcripts was seen with MOI 0.1 over time starting at 24 hpi. Infection of cells with MOI 0.01, although less compared to other MOIs, showed increasing levels of viral transcripts being made over time with the highest production of viral mRNA observed at 48 hpi (Fig. 1c). The VP16 and gD protein levels were also determined by immunoblotting. A similar trend was observed in the viral transcript levels. Viral protein levels were the highest at 24 hpi with MOI 1. In contrast the protein levels diminished at 48 hpi. Infection with MOI 0.1 seemed to show an increasing trend of viral proteins (Fig. 1d,e). The above results indicate that even viral transcription and protein production depends on the MOI and also depends on the time post infection.

### Viral MOIs dictate the extent of HSV-1 replication and viral release

**Figure 2:**
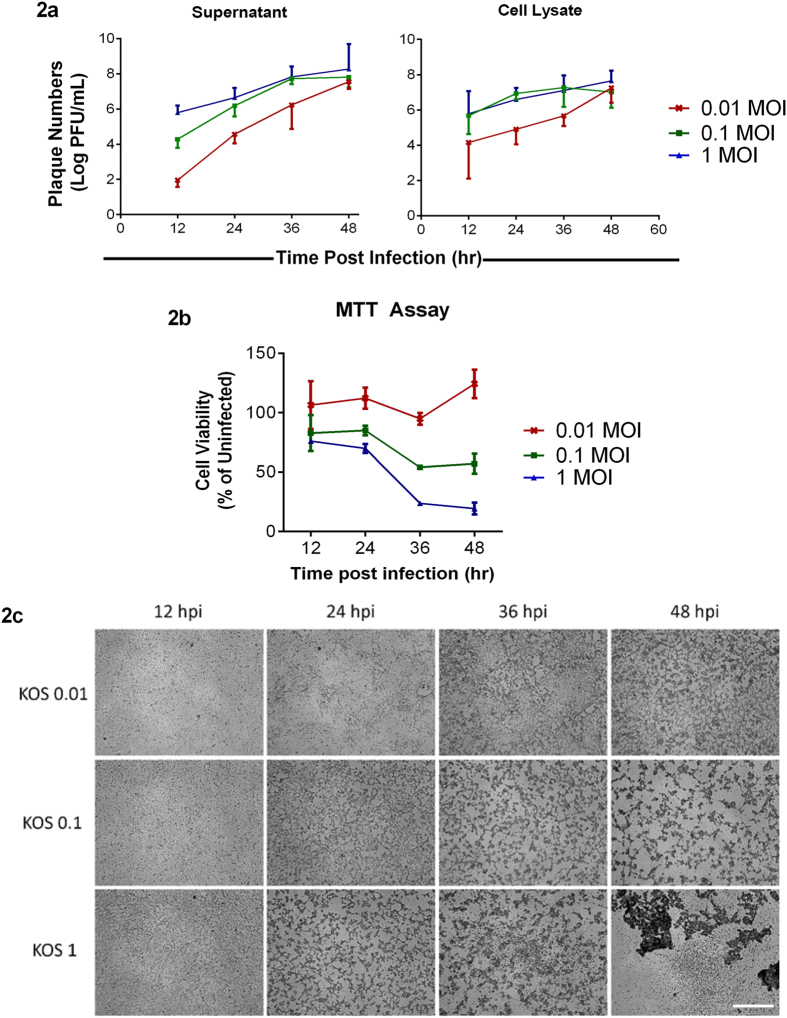
Initial titers determine the amount of virus released and cell viability. **(a)** Supernatant and cell lysates from HCE cells infected at different MOIs were collected at the indicated times and plaque assay was performed on Vero cells as mentioned under Materials and Methods. Significance was determined by Two-way ANOVA using Tukey’s correction. **Supernatant**: 12 hpi: 0.01 vs 0.1, **P < 0.01; 0.01 vs 1.0, ****P < 0.0001; 0.1 vs 1.0, *P < 0.05. 24 hpi: 0.01 vs 0.1, *P < 0.05; 0.01 vs 1.0, **P < 0.01; 0.1 vs 1.0, ns. 36 hpi: 0.01 vs 0.1, *P < 0.05; 0.01 vs 1.0, *P < 0.05; 0.1 vs 1.0, ns. 48 hpi: 0.01 vs 0.1, ns; 0.01 vs 1.0, ns; 0.1 vs 1.0, ns. **Cells**: 12 hpi: 0.01 vs 0.1, ns; 0.01 vs 1.0, ns; 0.1 vs 1.0, ns. 24 hpi: 0.01 vs 0.1, ns; 0.01 vs 1.0, ns; 0.1 vs 1.0, ns. 36 hpi: 0.01 vs 0.1, ns; 0.01 vs 1.0, ns; 0.1 vs 1.0, ns. 48 hpi: 0.01 vs 0.1, ns; 0.01 vs 1.0, ns; 0.1 vs 1.0, ns. **(b)** A standard MTT assay was performed on infected HCE cells at the indicated time points. Color was read at 560 nm. Two-way ANOVA with Tukey’s correction was performed to determine significance. 12 hpi: 0.01 vs 0.1, **P < 0.01; 0.01 vs 1.0, ***P < 0.001; 0.1 vs 1.0, ns. 24 hpi: 0.01 vs 0.1, ***P < 0.001; 0.01 vs 1.0, ****P < 0.0001; 0.1 vs 1.0, ns. 36 hpi: 0.01 vs 0.1, ****P < 0.0001; 0.01 vs 1.0, ****P < 0.0001; 0.1 vs 1.0, ***P < 0.001. 48 hpi: 0.01 vs 0.1, ****P < 0.0001; 0.01 vs 1.0, ****P < 0.0001; 0.1 vs 1.0, ****P < 0.0001 **(c)** Representative bright field images of HCE cells at indicated times post-infection. Scale bar for all images: 500 μm.

To determine how virus spread occurs in different MOIs, we performed a plaque assay^21^. We consistently observed that infectious virus particles in the supernatant were higher with MOI 1 infection at various times post infection (Fig. 2a). Virus titers from the cell lysates suggest that MOI 0.1 has higher infectious virus particles compared to other MOIs except at 12 and 48 hpi (Fig. 2a); this indicates that cells with MOI 1 infection produce virus particles at a faster rate and egress faster too. Hence, while the level of infectious viruses in the cells is lower for MOI 1, their levels are higher in the supernatant. These results are in line with our above results indicating that even virus spread depends on the initial virus inoculum.

### Viability of HSV-1 infected cells also depends on the initial MOI

Because HSV-1 infection *in vitro* becomes lytic and leads to cell death^22^, we assessed cell viability via the MTT assay. We observed that the viability of cells decreased as infection progressed and consistently a higher amount of dead cells was observed in cells infected with MOI 1 followed by MOI 0.1 at all times post infection (Fig. 2b). We also captured brightfield images of infected cells at the indicated times, and the images are consistent with our MTT results (Fig. 2c).

### Intracellular cytokine levels may modulate the rate of infection

**Figure 3:**
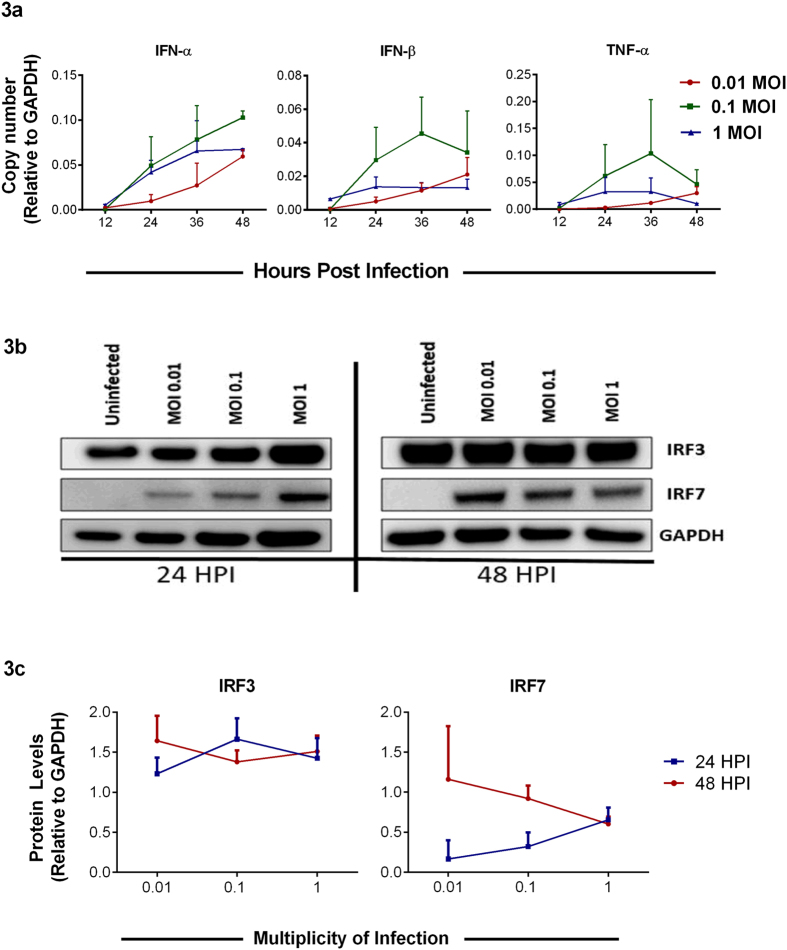
Viral dose-dependence of intracellular cytokine response. **(a)** Host cytokine transcripts were quantified by qRT-PCR from HCE cells at indicated times post-infection. Relative levels to GAPDH are shown. Two-way ANOVA was performed using Tukey’s correction. **IFN-α**: 12 hpi: 0.01 vs 0.1, ns; 0.01 vs 1.0, ns; 0.1 vs 1.0, ns. 24 hpi: 0.01 vs 0.1, *P < 0.05; 0.01 vs 1.0, ns; 0.1 vs 1.0, ns. 36 hpi: 0.01 vs 0.1, **P < 0.01; 0.01 vs 1.0, ns; 0.1 vs 1.0, ns. 48 hpi: 0.01 vs 0.1, *P < 0.05; 0.01 vs 1.0, ns; 0.1 vs 1.0, **P < 0.01. **IFN-β**: 12 hpi: 0.01 vs 0.1, ns; 0.01 vs 1.0, ns; 0.1 vs 1.0, ns. 24 hpi: 0.01 vs 0.1, *P < 0.05; 0.01 vs 1.0, ns; 0.1 vs 1.0, ns. 36 hpi: 0.01 vs 0.1, ***P < 0.001; 0.01 vs 1.0, ns; 0.1 vs 1.0, **P < 0.01. 48 hpi: 0.01 vs 0.1, ns; 0.01 vs 1.0, ns; 0.1 vs 1.0, **P < 0.01. **TNF-α**: 12 hpi: 0.01 vs 0.1, ns; 0.01 vs 1.0, ns; 0.1 vs 1.0, ns. 24 hpi: 0.01 vs 0.1, ns; 0.01 vs 1.0, ns; 0.1 vs 1.0, ns. 36 hpi: 0.01 vs 0.1, **P < 0.01; 0.01 vs 1.0, ns; 0.1 vs 1.0, *P < 0.05. 48 hpi: 0.01 vs 0.1, ns; 0.01 vs 1.0, ns; 0.1 vs 1.0, ns. **(b)** A representative immunoblot of IRF3 and IRF7 from HCE cells in 6 well plates infected with different MOIs and harvested at indicated times is shown. **(c)** Protein expression levels for the representative blot were quantified using ImageJ software. These experiments were performed in duplicates.

HSV-1 is known to drive host cell production of various cytokines and chemokines upon infection^7^. With this in mind, we were interested in determining how the production of these factors varies with viral dosage in our system. Interestingly, while the cells infected with an intermediate viral dose at MOI 0.1 consistently showed higher levels of the selected cytokine transcripts as demonstrated by qRT-PCR analysis, the cells infected with the lowest MOI showed a steady increase over time (Fig. 3a). On further probing, the interferon regulatory factors (IRFs) −3 and −7, major transcription factors involved in generating type 1 interferon responses upon HSV-1 infection, we observed that while IRF3 protein levels showed minor changes among different MOIs, IRF7 protein levels were found to increase at later times post infection and were the highest at MOI 0.01 (Fig. 3b,c). These findings suggest that epithelial cells are most effective at generating a potent cytokine response when allowed ample time to react to viral products, rather than subjected to the highly lytic nature of a large initial viral dose.

### *Ex vivo* cultures of the cornea restrict HSV-1 infection

**Figure 4:**
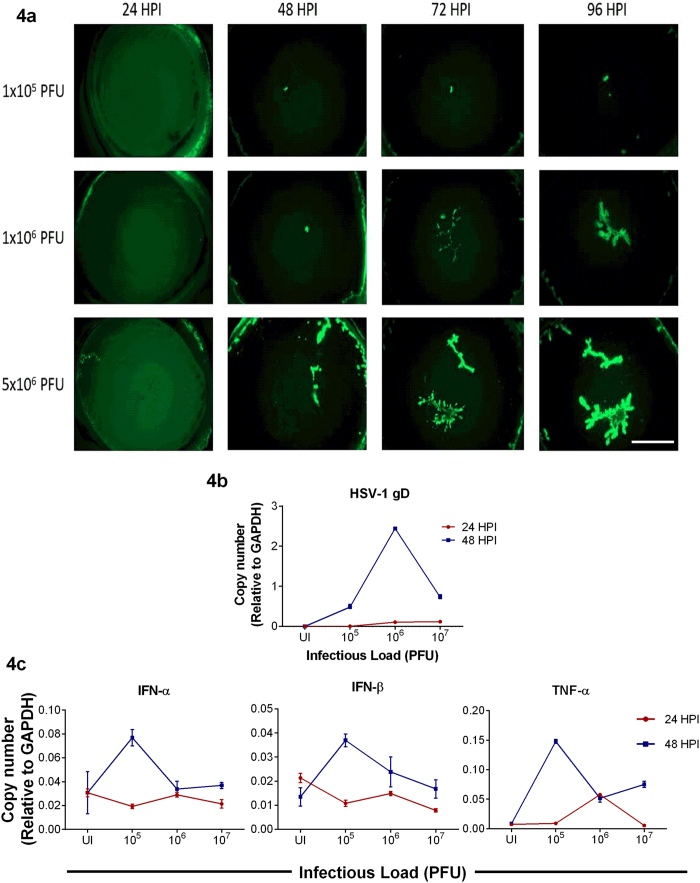
Viral spread in cultured corneas. **(a)** Representative images of porcine corneas showing the spread of HSV-1 17-GFP virus at indicated virus inoculums and times post-infection. Scale bar for all images: 1000 μm. **(b)** qRT-PCR analysis of viral gD transcripts from porcine corneas at indicated times are shown. Relative levels to GAPDH are shown. Two-way ANOVA was performed using Tukey’s correction. 24 hpi: UI vs 10^5^, 10^6^ vs 10^7^: ns; UI vs 10^6^, 10^5^ vs 10^6^, 10^5^ vs 10^7^: **P < 0.01; UI vs 10^7^: ***P < 0.001. 48 hpi: UI vs 10^6^, 10^6^ vs 10^7^, 10^5^ vs 10^6^, UI vs 10^5^, UI vs 10^7^, 10^5^ vs 10^7^: ****P < 0.0001 **(c)** qRT-PCR analysis of intracellular cytokine transcripts from porcine corneas are shown. Relative levels to GAPDH are shown. Two-way ANOVA was performed using Sidak’s correction. **TNF-α**: 24 hpi: UI vs 10^6^, 10^6^ vs 10^7^, 10^5^ vs 10^6^: ****P < 0.0001. UI vs 10^5^, UI vs 10^7^, 10^5^ vs 10^7^: ns; 48 hpi: UI vs 10^6^, 10^6^ vs 10^7^, 10^5^ vs 10^6^, UI vs 10^5^, UI vs 10^7^, 10^5^ vs 10^7^: ****P < 0.0001. **IFN-α**: 24 hpi: UI vs 10^6^, 10^6^ vs 10^7^, 10^5^ vs 10^6^, UI vs 10^5^, UI vs 10^7^, 10^5^ vs 10^7^: ns. 48 hpi: UI vs 10^6^, UI vs 10^7^, 10^6^ vs 10^7^: ns. UI vs 10^5^, 10^5^vs 10^6^, 10^5^ vs 10^7^: ****P < 0.0001. **IFN-β**: 24 hpi: UI vs 10^6^, 10^5^ vs 10^6^, 10^5^ vs 10^7^, 10^6^ vs 10^7^: ns. UI vs 10^5^, **P < 0.01. UI vs 10^7^, ***P < 0.001. 48 hpi: UI vs 10^7^, 10^6^ vs 10^7^: ns. UI vs 10^5^, 10^5^ vs 10^7^: ****P < 0.0001. UI vs 10^6^: **P < 0.01. 10^5^ vs 10^6^: ***P < 0.001.

**Figure 5:**
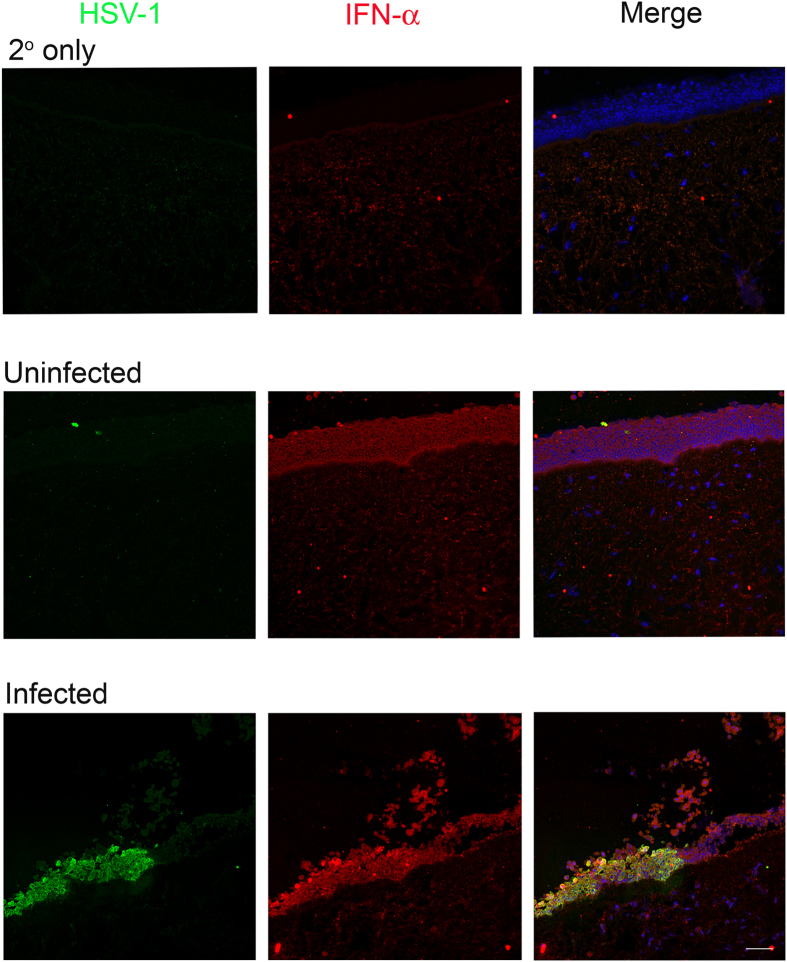
Levels of Interferon-α (IFN-α) in porcine corneal tissues. The cultured corneas were infected with 10^7^ PFU of HSV-1 (17-GFP) or mock infected. Representative IHC sections show the presence of HSV-1 (in green) and IFN-α (in red) in corneal epithelial cells (stained with DAPI in blue). Scale bar for all images: 50 μm.

Finally, in order to extend and also to verify our *in vitro* observations we employed a well-established HSV-1 *ex vivo* infection model^11^,^13^,^23^,^24^. Similar to the *in vitro* studies, the *ex vivo* model has not been extensively studied and very little is known about the dendritic nature of HSV-1 spread in the cultured tissue. In order to be able to see the spread of the virus we used a green fluorescence protein (GFP) expressing HSV-1 virus for the imaging part of our studies^25^. Porcine corneas were infected with HSV-1 17-GFP at indicated plaque forming units (PFU) and imaged at an interval of 24 h for a total period of 96 h. Initially, in all cases no GFP positive viral colonies were visible. As discussed next, starting at 24 hpi green colonies appeared in some cases but not in all. To our surprise, at no time during the 96 h period GFP virus spread or colony formation was noticed in the corneas infected with 10^4^ PFU (data not shown). Only a noticeably small viral spread was observed in the corneas infected with 10^5^ PFU; however, corneas infected with 10^6^ PFU showed larger and visible viral colony formation at 96 hpi. Corneas infected with 5 × 10^6^ or higher PFUs consistently showed viral spread and colony formation in a dendritic manner, which is consistent with clinical reports of HSV-1 dendritic ulcers^10^ (Fig. 4a). The presence of infection in these corneas was further confirmed by extracting RNA from epithelial cells and determining gD viral transcript levels at the indicated times (Fig. 4b). While at 24 hpi, the amount of gD transcripts was similar in 10^6^ and 10^7^ PFUs, we observed that gD mRNA levels were generally higher at 10^6^ PFU compared to other PFUs. This result is in contrast to the GFP images that we captured where we observe more infection at the highest PFU but in line with our *in vitro* results where we saw higher levels of viral transcripts at the intermediate MOI. We also probed for IFN-α, IFN-β and TNF-α transcripts (Fig. 4c). At 24 hpi, we did not see differences between different PFUs. Interestingly, at 48 hpi, 10^5^ PFU showed the highest levels of interferon transcripts possibly explaining the lack of infection at this PFU. Transcript levels were determined from a pool of epithelial cells containing infected and uninfected cells. To determine whether infected epithelial cells produce cytokines, we performed immunohistochemistry on uninfected and infected corneal tissue sections. We observed that infected epithelial cells produced more IFN-α compared to uninfected epithelial cells (Fig. 5). Overall, contrary to the cultured cells, the *ex vivo* model appears to restrict virus replication at or below 10^4^ PFU. The virus spread in the *ex vivo* model resembles the clinical pathology of the herpes simplex virus, whereas the *in vitro* model provides an excellent way to study fundamental virus-host interactions.

## Discussion

Our results provide novel insights on the unique aspects of HSV-1 infection of cultured HCE cells and cultured corneas. Numerous studies in the past have investigated the role of viral proteins in eliciting immune response and exploitation of the same for therapeutic purposes^26^,^27^,^28^,^29^. However, no studies have elucidated the role of host-cell response during a productive HSV-1 infection of the cornea or the cells of the cornea. In this study, we have integrated various aspects of disease progression (entry, replication, and egress) to portray a comprehensive outlook of intracellular activity (transcriptional) post HSV-1 infection using *in vitro* and *ex vivo* models.

Viral entry is facilitated by gB, gD, gH and gL, essential viral glycoproteins, attaching to host cell surface receptors: HVEM, Nectin-1, -2 and 3-OS heparan sulfate during primary infection^17^,^30^. Following this, virions fuse with the plasma membrane and are endocytosed by retrograde transport. We found that viral entry is dose dependent (Fig. 1a,b). These results suggest that increased MOIs have a larger load of the virus entering cells, and productive infection rate varies according to the initial dosage.

Following entry, virus undergoes adsorption, during which the capsid DNA is translocated into the nucleus. Our results show that VP16 mRNA transcripts peak at 24 hpi and diminish over time (Fig. 1c); this could be attributed to the biphasic role of VP16 in early and late stages of viral lytic cycle^31^,^32^. The VP16 transcript levels correlate with immunoblotting results for VP16 where we observe an increasing trend for cells infected with lower MOIs - 0.01 and 0.1 and a decreasing trend in the higher MOI – 1 (Fig. 1d,e). Our results further show an increase in gD mRNA transcript in all the samples over time (Fig. 1c). However, the gD protein levels immunoblotted over the same period show a decrease (Fig. 1d,e). This could be attributed to the following reasons as infection proceeds to the later stages of infection: (i) the progeny gets assembled and egresses out of the cell as supported by our plaque assay data (Fig. 2a). Thus, the gD protein levels are found to be lower at 48 hpi. (ii) Lytic cycle triggers and the infection results in the death of cells. This finding is confirmed in our cell viability results (Fig. 2b) where we observe that at 48 hpi cells are not only unhealthy but are no longer adhered to the dishes. These results can also be correlated with the cytokine responses noted for these cells, which shows an increase in IFN-α and IFN-β for those infected with lower MOI rather those infected with a higher MOI (Fig. 3a). This could be attributed to suppressed cytokine production and activity in cells infected with a greater MOI rather than those infected with lower MOI. In a way, it is easy to speculate that cells infected with a lower MOI have a greater chance at fighting viral infection than those infected with a higher MOI. A balance between viral replication and intracellular cytokine response could decide the fate of a productive infection, which in turn can allow virus growth for longer periods of time rather than lyse the cell.

In order to understand the effects of HSV-1 infection of HCE cells further, experiments to determine the total number of infectious virions egressing the cell were performed by plaque assays using supernatants and cell lysates of infected cells at multiple time points. Our results indicated a ten fold increase in viral egress at every time point for every MOI. However, the total number of virions per cell seem to reach a certain threshold value before their numbers start to decrease. This threshold value for the number of virions exiting the cells could be attributed to the total available resources for the virus inside the cell to generate a number of virions. At 36 hpi (Fig. 2a), the trend for MOI 0.1 and 1 exhibits complete reversal. We see fewer viruses in the cells at MOI 1 compared to MOI 0.1 because most of the viruses are likely released into the media and hence the change in viral titers from cell lysates. For reasons poorly understood, we also observed that while the infectious virus titer increases over time for MOI 0.1, it drops at 48 hpi. Future experiments will be designed to investigate such changes.

Various factors have been known to contribute to the overall viability of cells post infection^33^,^34^,^35^,^36^,^37^. In the past, studies have examined the role of autophagy, mitophagy, and interferon in the cell activity^38^. We found that viability of cells increases as the viral dosage per cell decreases by one order of magnitude (Fig. 2b). As virions enter viable cells, viral protein *vhs*, or U_L_41, interacts with host gene IRFs and suppresses the interferon response of the respective cell. Th *vhs* protein is known to deplete the levels of Jak1 and TNF-R1^39^, protein kinases that are intermediates in the interferon pathways. We thus probed for endogenous cytokines and IRF levels (Fig. 3) to correlate them with cell viability. With MOI 1 infection, since each cell is infected with one infectious unit, the virus efficiently completes its lytic cycle in a shorter period, and hence lower viablity is observed in cells infected at this MOI. On the other hand, MOI 0.1 has only one in ten cells infected with infectious units, so in theory, it might take much longer for all cells to undergo lytic cycle. As a result, cells infected with MOI 0.1 have longer viability and allow productive infection to last longer. In MOI 0.01, one in hundred cells are infected with virion and the rest cells are viable to produce cytokines to suppress and limit the infection spread; due to the limitation mentioned above, MOI 0.01 infection lasts for very short period resulting in suppression of productive cycle *in vitro*. As clearly seen (Fig. 2c), at 48 hpi, the majority of cells infected with MOI 1 are dead, which explains why no signal was detected in the cellular transcript expression of 48 hpi.

*In vitro* models have a limitation in that the cells cannot be cultured for longer periods of time as they not only require frequent media change but the cells eventually become overconfluent and start to detach or die. To better study the effects of long term infection, we employed an *ex vivo* model by utilizing porcine corneas. The corneas were infected with the indicated PFUs in order to examine the cellular response to infection in this unique model. We observed no or less viral spread and colony formation at the lower inoculums and a good amount of viral spread and colony formation at higher inoculums showing a unique dendritic pattern that mimics the dendritic pathologies of HSV-1 ulcers in the cornea^10^ (Fig. 4a). Infection of corneas at 10^6^ PFU showed the highest levels of gD transcripts (Fig. 4b). We are currently not sure of this finding and future studies to understand this trend will be planned. Our finding that the lowest inoculum of 10^4^–10^5^ PFUs displayed no productive infection is interesting because low viral dosages in the *in vitro* model showed good signs of infection. This observation supports the notion that the eye is an immunoprivileged organ that possesses intrinsic mechanisms to protect the eye against viral infections. The architecture of the corneal epithelial cells may contribute to one part of this intrinsic mechanism. The *in vitro* model has non-polarized cells that either lack or have loose cell-to-cell junctions that allows the virus to infect the apical and basal surfaces resulting in more spread between the cells via viral induced cell fusion or by infection followed by egress. On the other hand, porcine corneas have polarized cells with tight cell-to-cell junctions and the cells are stacked in a 3D structure. Since only the apical surface is exposed to the virus and it has been shown that HSV preferentially accesses the apical surface via nectin-1^40^, virus infection and spread is restricted. Reports have also suggested the presence of resident immune cells that infiltrate the cornea upon HSV-1 infection and provide protection^41^ and these resident immune cells could also contribute to another part of the intrinsic mechanism. However in this study, we have not determined the presence of resident immune cells due to lack of antibodies against porcine immune cells of interest and moreover because there is no systemic immune system present, infiltration of these resident cells will be restricted. Another factor that could contribute to the intrinsic mechanism is the role of the host restriction factor: tetherin in restricting HSV-1 infections^42^; future experiments will be designed to investigate this. To examine whether intracellular cytokines play a role in the intrinsic mechanism, we examined the transcripts via qRT-PCR (Fig. 4c). The *ex vivo* model provides an advantage of specifically checking for intracellular cytokine levels as there is no active immune system present. We observed that while at 24 hpi there was no specific pattern identified, at 48 hpi, at lower PFUs the interferon transcripts were higher compared to higher PFUs. Although the interferon transcripts *in vitro* and *ex vivo* are produced at similar levels, the reasons mentioned above along with interferon responses may be efficient in curbing viral infection in the *ex vivo* model compared to the *in vitro* model at lower viral dosage. The interferon transcipts determined do not distinguish its origin of production between infected and uninfected cells. We therefore performed IHC to identify whether infected cells produce interferons (Fig. 5). The representative stained sections show that infected corneal tissues have more IFN-α on the epithelium compared to uninfected corneal tissues which is consistent with the existing literature suggesting that HSV-1 infection induces IFN production^42^. More studies will be needed to precisely determine the complex interplay between viral dose for infection and the cytokine and other local responses generated by the cells of the cornea.

In conclusion, cultured HCE cells were productively infected by all the viral dosages examined. However, the *ex vivo* cultures did not result in a productive infection below 1 × 10^5^ PFU indicating an apparent restriction to infection at or below this threshold level. It is likely that the unique architecture of the cornea as well as a concerted action of resident immune cells, host restriction factors and interferon levels can control infection in the cornea; this control does not require help from the systemic immune system, which is absent when the corneas are cultured *ex vivo* and infected with HSV-1. Our findings will guide many future basic science studies and drug discovery research on HSV-1 infection of the cornea.

## Materials and Methods

### Cells, viruses and antibodies

Human Corneal Epithelial cells (RCB1834 HCE-T) was obtained from Kozaburo Hayashi (National Eye Institute, Bethesda, MD) and was cultured in MEM (Life Technologies, Carlsbad, CA) with 10% fetal bovine serum (FBS, Sigma-Aldrich, St. Louis, MO) and 1% penicillin/streptomycin (P/S, Life Technologies). The African green monkey kidney (VERO) cell lines were obtained from Dr. Patricia G. Spear (Northwestern University, Chicago, IL) and cultured in DMEM (Life Technologies) with 10% FBS and 1% P/S.

HSV-1 KOS (wild type, gL-86, 17-GFP and K26-GFP^25^) strains were used in this study. The strains were propagated and titered on VERO cells. Aliquots of the virus were stored at −80 °C.

VP16 (1:500), GAPDH (1:1000) (SantaCruz Biotech, Santa Cruz, CA), gD and ICP0 (1:1000) (Abcam, Cambridge, United Kingdom), IRF-3 and -7 (1:1000) (Cell Signaling Technology, Danvers, MA) antibodies were used in this study.

### Infection

Cells were counted using the Countess^TM^ Automated Cell Counter (Invitrogen, Carlsbad, CA) and accordingly MOI calculations were performed. Cells were incubated with virus in phosphate-buffered saline (PBS, Life Technologies, Carlsbad, CA) supplemented with 0.1% glucose (Fisher, Waltham, MA) and 1% fetal bovine serum (PBS-GCS) at 37 °C -5% CO_2_. Two hours post infection, the virus solution was removed and fresh culture media was added to the cells.

### Entry Assay

HCE cells in 96 well plates were infected with recombinant HSV-1 gL86 virus at indicated MOIs. At 6 hpi, ONPG solution (3 mg/mL ONPG (Invitrogen), 0.05% NP-40 (USB Corp., Cleveland, OH) in PBS) was added following a wash with PBS and the plate was incubated at 37 °C for 30–120 min. The color developed was read on a microplate reader (Tecan GENious Pro, Mannedorf, Switzerland) at 410 nm.

### Quantitative-RT PCR (qRT-PCR) Assay

RNA was extracted from the cells using TRIzol (Life Technologies) according to manufacturer’s described protocol. 2 μg RNA was then reverse transcribed into cDNA using High Capacity cDNA Reverse Transcription Kit (Applied Biosystems, Foster City, CA). Real-time quantitative PCR was performed using Fast SYBR Green Master Mix using QuantStudio 7 Flex system (Applied Biosystems). The primers used in this study are listed in Tables 1, 2 and 3 in a 5′-3′ manner.

### Immunoblotting

Cells were lysed in radio-immunoprecipitation assay (RIPA, Sigma-Aldrich, St. Louis, MO) buffer according to the manufacturer’s protocol. After gel electrophoresis, transfer to a PVDF membrane was performed using the iBlot gel transfer device (ThermoFisher Scientific, Waltham, MA). After the transfer, the membrane was blocked in 5% milk/TBS (Bio-Rad, Hercules, CA)-Tween 20 (Fisher) for 1 hour followed by incubation with primary antibodies overnight at 4 °C. Incubation with corresponding horseradish peroxidase-conjugated secondary antibodies for 1 hour followed and subsequently the SuperSignal West Femto maximum sensitivity substrate (Thermo Scientific) was added. The bands were visualized and imaged using ImageQuant LAS 4000 imager (GE Healthcare Life Sciences, Pittsburg, PA). The intensity of the bands were analyzed using ImageJ analysis software.

### MTT assay and brightfield imaging

HCE cells in 96 well plates were infected with HSV-1 KOS at the indicated MOIs. At 12, 24, 36 and 48 hpi, cells were washed with PBS and 0.5 mg/mL MTT reagent in culture media (Promega, Madison, WI) was added and incubated for 2 hrs at 37 °C. After incubation, the color developed was read on a microplate reader (TECAN) at 560 nm. Similarly, to visualize viability of cells, HCE cells in 12 well plates were imaged under 4x objective using an Axiovert 200 (Carl Zeiss, Germany).

### Plaque assay

Monolayers of HCE were plated on 6 well plates and infected with HSV-1 KOS virus at MOI of 1, 0.1 and 0.01. Media and cell lysates were collected at 12, 24, 36 and 48 hpi. For 12 and 24 hpi, the supernatant were collected and centrifuged to pellet out the debris that was discarded. For 36 and 48 hpi, because the cells were no longer adhered to the dishes, we collected the cells and media together, centrifuged them and then separated out the supernatant and cells. The supernatant and cell lysates serially diluted and incubated with Vero cells to determine viral titers. After 2 hour incubation, the Vero cells were incubated with complete DMEM containing 1% methylcellulose (Fisher) for 72 hours. To visualize and count plaques, cells were fixed with 100% methanol and stained with crystal violet solution.

### *Ex vivo* corneal infection and qRT-PCR analysis

Corneal tissues were obtained from Park Packing, Inc., Chicago, IL. Whole eyes were removed immediately after sacrificing the porcine and brought to the lab in a box containing ice packs. The corneal tissues were excised from the whole eye and incubated in serum-free MEM supplemented with 5% antibiotic-antimycotic (Gibco) and 1% insulin-transferrin-sodium-selenite (Sigma-Aldrich) for 1 hour at 37 °C. Prior to being excised from the eye, the corneal epithelium was punctured once with a 30G needle. The excised corneas were rinsed in PBS with 5% antibiotic-antimycotic solution before they were placed epithelial side up in a 12 well plate. Culture media was inoculated with the appropriate amount of 17-GFP stock for each PFU sample group and corneas were submerged in the inoculum for 24 hours. After 24 hours, the infection media was removed, corneas were washed with PBS, and fresh culture media was added dropwise until the limbus was covered. At 24-hour intervals post infection, the corneas were imaged using a SteREO Discovery.V20 (Carl Zeiss) at a magnification of 12.5X for the presence of GFP virus. Media was changed every 48 hours. Similarly, at the indicated times post infection, using a sterile surgical blade epithelial cells were gently scraped off the cornea and collected in TRIzol (Life Technologies). RNA extraction and qRT-PCR was performed as described above.

### Immunohistochemistry

Porcine corneas were either uninfected or infected with 10^7^ PFU of 17-GFP virus as mentioned above. At 48 hpi the corneas were processed for IHC as follows: corneas were washed with ice-cold PBS and embedded in optimal cutting temperature (OCT, Tissue-Tek, Sakura, Zoeterwoude, The Netherlands) compound and flash frozen on dry ice. 5 μm thick sections were cut using the Cryostar NX50 (Thermo Scientific) microtome and fixed in cold Acetone (Fisher) at −20 °C for 10 minutes. The sections were then washed with PBS, blocked in 1% BSA for 1 hr and then incubated with a dual cocktail of primary antibodies against mouse IFN-α (PBL Assay Science, Piscataway, NJ) and rabbit HSV-1 (Abcam) in 1% BSA at 4 °C overnight. As a control, sections were incubated with 1% BSA only. Following washes, the sections were then incubated with Anti-mouse Cy3 and Anti-rabbit FITC (Life Technologies) for 1 hr at room temperature before washing the sections and mounting them in a media containing DAPI (VectaShield; Vector Laboratories, Burlingame, CA, USA). The sections were imaged under a confocal microscope (Zeiss 710, Carl Zeiss) at 20x objectives.

### Statistical Analysis

All experiments were performed in triplicates, unless otherwise stated. Data shown are means ± SD. Appropriate statistical significance tests were performed using GraphPad Prism (GraphPad Software, La Jolla, CA, USA). *Asterisks* denotes a significant difference; *P < 0.05, **P < 0.01, ***P < 0.001, ****P < 0.0001, ns- non-significant.

## Additional Information

**How to cite this article**: Thakkar, N. *et al*. Cultured corneas show dendritic spread and restrict herpes simplex virus infection that is not observed with cultured corneal cells. *Sci. Rep.* **7**, 42559; doi: 10.1038/srep42559 (2017).

**Publisher's note:** Springer Nature remains neutral with regard to jurisdictional claims in published maps and institutional affiliations.

## Figures and Tables

**Table 1:** Human Primer Sequence.

Human Primers	Sequences* (F: Forward, R: Reverse)
GAPDH	**F**: CACCACCAACTGCTTAGCAC
	**R**: CCCTGTTGCTGTAGCCAAAT
IFN-β	**F**: CTCCACTACAGCTCTTTCCAT
	**R**: GTCAAAGTTCATCCTGTCCTT
IFN-α	**F**: GATGGCAACCAGTTCCAGAAG
	**R**: AAAGAGGTTGAAGATCTGCTGGAT
TNF-α	**F**: AGCCCATGTTGTAGCAAACCC
	**R**: GGACCTGGGAGTAGATGAGGT

List of primers used for qRT-PCR.

**Table 2:** Porcine Primer Sequence.

Porcine Primers	Sequence* (F: Forward, R: Reverse)
GAPDH	**F**:TACACTGAGGACCAGGTTGTG
	**R**: TTGACGAAGTGGTCGTTGAG
IFN-β	**F**: ACTGGCTGGAATGAAACCGT
	**R**: AATGGTCATGTCTCCCCTGG
IFN-α	**F**: CCAACCTCAGCCTTCCTCAC
	**R**: TCCTCATTTGTGCCAGGAGC
TNF-α	**F**: GGCCCAAGGACTCAGATCAT
	**R**: CTGTCCCTCGGCTTTGACAT

List of primers used for qRT-PCR.

**Table 3:** HSV-1 Primer Sequences.

HSV-1 Primers	Sequence* (F: Forward, R: Reverse)
VP16	**F:** TCGGCGTGGAAGAAACGAGAGA
	**R:** CGAACGCACCCAAATCGACA
gD	**F:** TACAACCTGACCATCGCTTC
	**R**: GCCCCCAGAGACTTGTTGTA

List of primers used for qRT-PCR.

## References

[b1] Shukla D. & Spear P. G. Herpesviruses and heparan sulfate: an intimate relationship in aid of viral entry. J. Clin. Invest. 108, 503–510 (2001). 11518721 10.1172/JCI13799 PMC209412

[b2] Akhtar J. & Shukla D. Viral entry mechanisms: cellular and viral mediators of herpes simplex virus entry. FEBS J. 276, 7228–7236 (2009). 19878306 10.1111/j.1742-4658.2009.07402.x PMC2801626

[b3] Whitley R. J. & Roizman B. Herpes simplex viruses: is a vaccine tenable? J. Clin. Invest. 110, 145–151 (2002). 12122103 10.1172/JCI16126 PMC151069

[b4] Whitley R. J. & Kimberlin D. W. Herpes simplex encephalitis: children and adolescents. Semin. Pediatr. Infect. Dis. 16, 17–23 (2005). 15685145 10.1053/j.spid.2004.09.007

[b5] Farooq A. V. & Shukla D. Herpes simplex epithelial and stromal keratitis: an epidemiologic update. Surv. Ophthalmol. 57, 448–462 (2012). 22542912 10.1016/j.survophthal.2012.01.005 PMC3652623

[b6] Rowe A. M. . Herpes keratitis. Prog. Retin. Eye Res. 32, 88–101 (2013). 22944008 10.1016/j.preteyeres.2012.08.002 PMC3529813

[b7] Gimenez F., Suryawanshi A. & Rouse B. T. Pathogenesis of herpes stromal keratitis - A focus on corneal neovascularization. Prog. Retin. Eye Res. 33, 1–9 (2013). 22892644 10.1016/j.preteyeres.2012.07.002 PMC3511644

[b8] Park P. J. . Corneal lymphangiogenesis in herpetic stromal keratitis. Surv. Ophthalmol. 60, 60–71 (2015). 25444520 10.1016/j.survophthal.2014.06.001 PMC4262646

[b9] Liesegang T. J. Herpes simplex virus epidemiology and ocular importance. Cornea 20, 1–13 (2001). 11188989 10.1097/00003226-200101000-00001

[b10] Shah A., Farooq A. V., Tiwari V., Kim M. J. & Shukla D. HSV-1 infection of human corneal epithelial cells: receptor-mediated entry and trends of re-infection. Mol. Vis. 16, 2476–2486 (2010). 21139972 PMC2994737

[b11] Park P. J., Antoine T. E., Farooq A. V., Valyi-Nagy T. & Shukla D. An investigative peptide-acyclovir combination to control herpes simplex virus type 1 ocular infection. Invest. Ophthalmol. Vis. Sci. 54, 6373–6381 (2013). 23989188 10.1167/iovs.13-12832 PMC3787657

[b12] Tiwari V., Liu J., Valyi-Nagy T. & Shukla D. Anti-heparan sulfate peptides that block herpes simplex virus infection *in vivo*. J. Biol. Chem. 286, 25406–25415 (2011). 21596749 10.1074/jbc.M110.201103 PMC3137111

[b13] Hadigal S. R. . Heparanase is a host enzyme required for herpes simplex virus-1 release from cells. Nat. Commun. 6, 6985 (2015). 25912399 10.1038/ncomms7985 PMC4413471

[b14] Agelidis A. M. & Shukla D. Cell entry mechanisms of HSV: what we have learned in recent years. Future Virol. 10, 1145–1154 (2015). 27066105 10.2217/fvl.15.85 PMC4822157

[b15] Farooq A. V., Valyi-Nagy T. & Shukla D. Mediators and mechanisms of herpes simplex virus entry into ocular cells. Curr. Eye Res. 35, 445–450 (2010). 20465436 10.3109/02713681003734841 PMC2902162

[b16] Srivastava R. . A Herpes Simplex Virus Type 1 Human Asymptomatic CD8+ T-Cell Epitopes-Based Vaccine Protects Against Ocular Herpes in a “Humanized” HLA Transgenic Rabbit Model. Invest. Ophthalmol. Vis. Sci. 56, 4013–4028 (2015). 26098469 10.1167/iovs.15-17074 PMC4477261

[b17] Spear P. G., Eisenberg R. J. & Cohen G. H. Three classes of cell surface receptors for alphaherpesvirus entry. Virology 275, 1–8 (2000). 11017782 10.1006/viro.2000.0529

[b18] Shukla D. . A novel role for 3-O-sulfated heparan sulfate in herpes simplex virus 1 entry. Cell 99, 13–22 (1999). 10520990 10.1016/s0092-8674(00)80058-6

[b19] Tiwari V. . Soluble 3-O-sulfated heparan sulfate can trigger herpes simplex virus type 1 entry into resistant Chinese hamster ovary (CHO-K1) cells. J. Gen. Virol. 88, 1075–1079 (2007). 17374750 10.1099/vir.0.82476-0

[b20] Jaishankar D., Yakoub A. M., Bogdanov A., Valyi-Nagy T. & Shukla D. Characterization of a proteolytically stable D-peptide that suppresses herpes simplex virus 1 infection: implications for the development of entry-based antiviral therapy. J. Virol. 89, 1932–1938 (2015). 25428865 10.1128/JVI.02979-14 PMC4300769

[b21] Yakoub A. M. & Shukla D. Autophagy stimulation abrogates herpes simplex virus-1 infection. Sci. Rep. 5, 9730 (2015). 25856282 10.1038/srep09730 PMC4929686

[b22] Flemington E. K. Herpesvirus lytic replication and the cell cycle: arresting new developments. J. Virol. 75, 4475–4481 (2001). 11312317 10.1128/JVI.75.10.4475-4481.2001 PMC114200

[b23] Drevets P. . The use of human cornea organotypic cultures to study herpes simplex virus type 1 (HSV-1)-induced inflammation. Graefes Arch. Clin. Exp. Ophthalmol. 253, 1721–1728 (2015). 26047535 10.1007/s00417-015-3073-4 PMC4573349

[b24] Alekseev O., Tran A. H. & Azizkhan-Clifford J. *Ex vivo* organotypic corneal model of acute epithelial herpes simplex virus type I infection. J. Vis. Exp. (69), e3631. doi, e3631 (2012). 23149439 10.3791/3631 PMC3514049

[b25] Desai P. & Person S. Incorporation of the green fluorescent protein into the herpes simplex virus type 1 capsid. J. Virol. 72, 7563–7568 (1998). 9696854 10.1128/jvi.72.9.7563-7568.1998 PMC110002

[b26] Tiwari V., Tarbutton M. S. & Shukla D. Diversity of heparan sulfate and HSV entry: basic understanding and treatment strategies. Molecules 20, 2707–2727 (2015). 25665065 10.3390/molecules20022707 PMC6272628

[b27] Antoine T. E., Park P. J. & Shukla D. Glycoprotein targeted therapeutics: a new era of anti-herpes simplex virus-1 therapeutics. Rev. Med. Virol. 23, 194–208 (2013). 23440920 10.1002/rmv.1740 PMC3661299

[b28] Bacon T. H., Levin M. J., Leary J. J., Sarisky R. T. & Sutton D. Herpes simplex virus resistance to acyclovir and penciclovir after two decades of antiviral therapy. Clin. Microbiol. Rev. 16, 114–128 (2003). 12525428 10.1128/CMR.16.1.114-128.2003 PMC145299

[b29] Chilukuri S. & Rosen T. Management of acyclovir-resistant herpes simplex virus. Dermatol. Clin. 21, 311–320 (2003). 12757254 10.1016/s0733-8635(02)00093-1

[b30] Oh M. J., Akhtar J., Desai P. & Shukla D. A role for heparan sulfate in viral surfing. Biochem. Biophys. Res. Commun. 391, 176–181 (2010). 19909728 10.1016/j.bbrc.2009.11.027 PMC2812628

[b31] Heine J. W., Honess R. W., Cassai E. & Roizman B. Proteins specified by herpes simplex virus. XII. The virion polypeptides of type 1 strains. J. Virol. 14, 640–651 (1974). 4369085 10.1128/jvi.14.3.640-651.1974 PMC355559

[b32] Mossman K. L., Sherburne R., Lavery C., Duncan J. & Smiley J. R. Evidence that Herpes Simplex Virus VP16 Is Required for Viral Egress Downstream of the Initial Envelopment Event. J. Virol. 74, 6287–6299 (2000). 10864638 10.1128/jvi.74.14.6287-6299.2000 PMC112134

[b33] Imperia P. S. . An *in vitro* study of ophthalmic antiviral agent toxicity on rabbit corneal epithelium. Antiviral Res. 9, 263–272 (1988). 3144240 10.1016/0166-3542(88)90057-5

[b34] Lass J. H., Langston R. H., Foster C. S. & Pavan-Langston D. Antiviral medications and corneal wound healing. Antiviral Res. 4, 143–157 (1984). 6476819 10.1016/0166-3542(84)90014-7

[b35] Mosmann T. Rapid colorimetric assay for cellular growth and survival: application to proliferation and cytotoxicity assays. J. Immunol. Methods 65, 55–63 (1983). 6606682 10.1016/0022-1759(83)90303-4

[b36] Chan K. Y., Cho P. & Boost M. Corneal epithelial cell viability of an *ex vivo* porcine eye model. Clin. Exp. Optom. 97, 337–340 (2014). 24438477 10.1111/cxo.12128

[b37] Choy E. P., To T. S., Cho P., Benzie I. F. & Choy C. K. Viability of porcine corneal epithelium *ex vivo* and effect of exposure to air: a pilot study for a dry eye model. Cornea 23, 715–719 (2004). 15448499 10.1097/01.ico.0000127475.29551.56

[b38] Dong X. & Levine B. Autophagy and viruses: adversaries or allies? J. Innate Immun. 5, 480–493 (2013). 23391695 10.1159/000346388 PMC3790331

[b39] Fields Bernard N., Knipe David M. & Howley Peter M. In Fields Virology (Wolters Kluwer Health/Lippincott Williams & Wilkins, Philadelphia, 2007).

[b40] Galen B., Cheshenko N., Tuyama A., Ramratnam B. & Herold B. C. Access to nectin favors herpes simplex virus infection at the apical surface of polarized human epithelial cells. J Virol. 88, 12209–18 (2006). 10.1128/JVI.01503-06 PMC1676285 17005657

[b41] Hamrah P. & Dana M. R. Corneal antigen-presenting cells. Chem Immunol Allergy. 92, 58–70(2007). 17264483 10.1159/000099254

[b42] Royer D. J. & Carr D. J. A STING-dependent innate-sensing pathway mediates resistance to corneal HSV-1 infection via upregulation of the antiviral effector tetherin. Mucosal Immunol. 9, 1065–1075 (2016). 26627457 10.1038/mi.2015.124 PMC4889566

